# Congenital Unilateral Syngnathia: A Report of a Rare Case

**DOI:** 10.7759/cureus.82839

**Published:** 2025-04-23

**Authors:** MR Haranadha Reddy, G V Reddy, Lavanya Ummadisingh, Aishwarya Ravikumar, Sheema Imtiaz

**Affiliations:** 1 Oral and Maxillofacial Surgery, Panineeya Mahavidyalaya Institute of Dental Sciences and Research Centre, Hyderabad, IND

**Keywords:** congenital maxillomandibular fusion, craniofacial anomaly, fused jaws, limited mouth opening, maxillomandibular synostosis, pediatric surgery, synechiae, syngnathia

## Abstract

Congenital maxillomandibular syngnathia is an uncommon craniofacial disorder, with limited documented cases in the literature. It involves abnormal fusion of the maxilla and mandible, resulting in restricted mouth opening and airway difficulties, and is associated with various syndromes. We report a case of a nine-year-old girl who has been unable to open her mouth since birth. This case highlights the critical need for precise diagnosis and timely surgical intervention to ensure the best possible outcomes for individuals with this condition.

## Introduction

Syngnathia is a congenital condition characterized by the fusion of the jaws, which can involve only soft tissue (synechiae) or, in some cases, bone (maxillomandibular synostosis). A total of 118 cases have been documented to date, with the first reported by Burket in 1936 [1]. The exact cause of syngnathia remains unclear. Clinically, it presents with limited mouth opening, leading to difficulties in feeding and breathing. Due to its rarity and the diverse ways it can manifest, managing this condition can be challenging and depends on the specific type and severity. This case report details a nine-year-old female patient presenting with bony fusion (synostosis) between the maxilla and mandible, accompanied by fibrous adhesions (synechiae) on the left side.

## Case presentation

**Figure 1:**
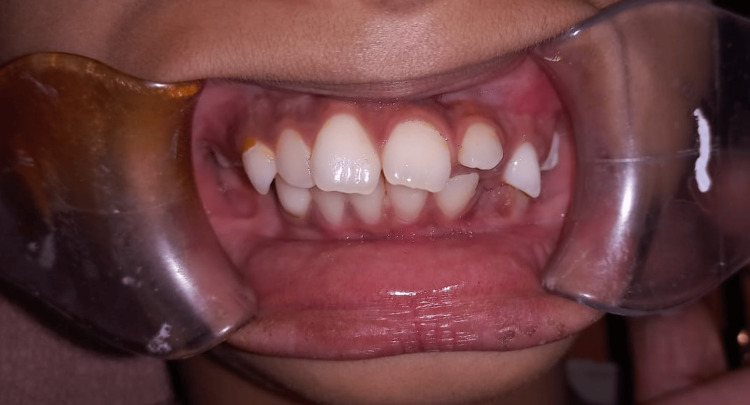
Zero mouth opening.

**Figure 2:**
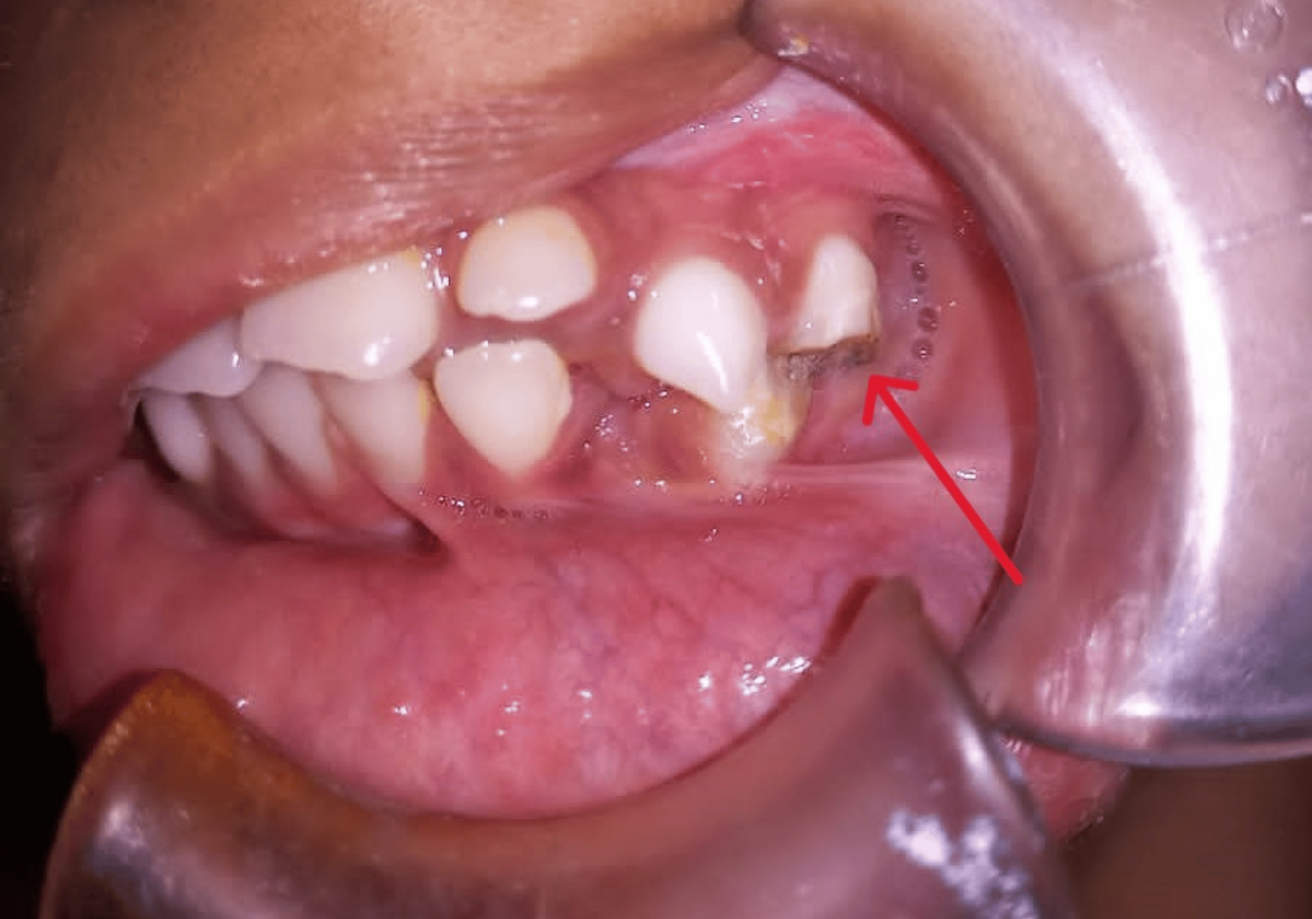
Fusion of mucosa can be seen on the left side. Clinically, no posterior limit can be found.

A nine-year-old female reported to the Department of Oral and Maxillofacial Surgery, Panineeya Mahavidyalaya Institute of Dental Sciences & Research Centre, Hyderabad, Telangana, India, with a chief complaint of inability to open her mouth since birth. Her parents also reported that the child had undergone a surgical intervention when she was one year old, and she managed to keep her mouth open for six months, after which the condition recurred. The patient is the family’s first child, and there is a similar anomaly in the younger sibling. The mother of the child had a healthy gestational period, and there was no history of illness, trauma, or drug use. On general physical examination, she showed no abnormalities. On extraoral examination, there were no temporomandibular joint (TMJ) movements bilaterally, and the mouth opening was 0 mm (Figure 1). The mandible appeared to be retruded. On intraoral examination, there was fair oral hygiene, and fusion of mucosa was seen distal to tooth 64 on the left side. On palpation, a bony hard mass joining the maxilla and mandible on the left side was noted, with clinically no posterior limit, and retromolar and tuberosity space obliteration seen on the same side (Figure 2). Teeth present were 11, 12, 53, 21, 22, 63, 64, 31, 32, 36, 41, 42, and 83.

**Figure 3:**
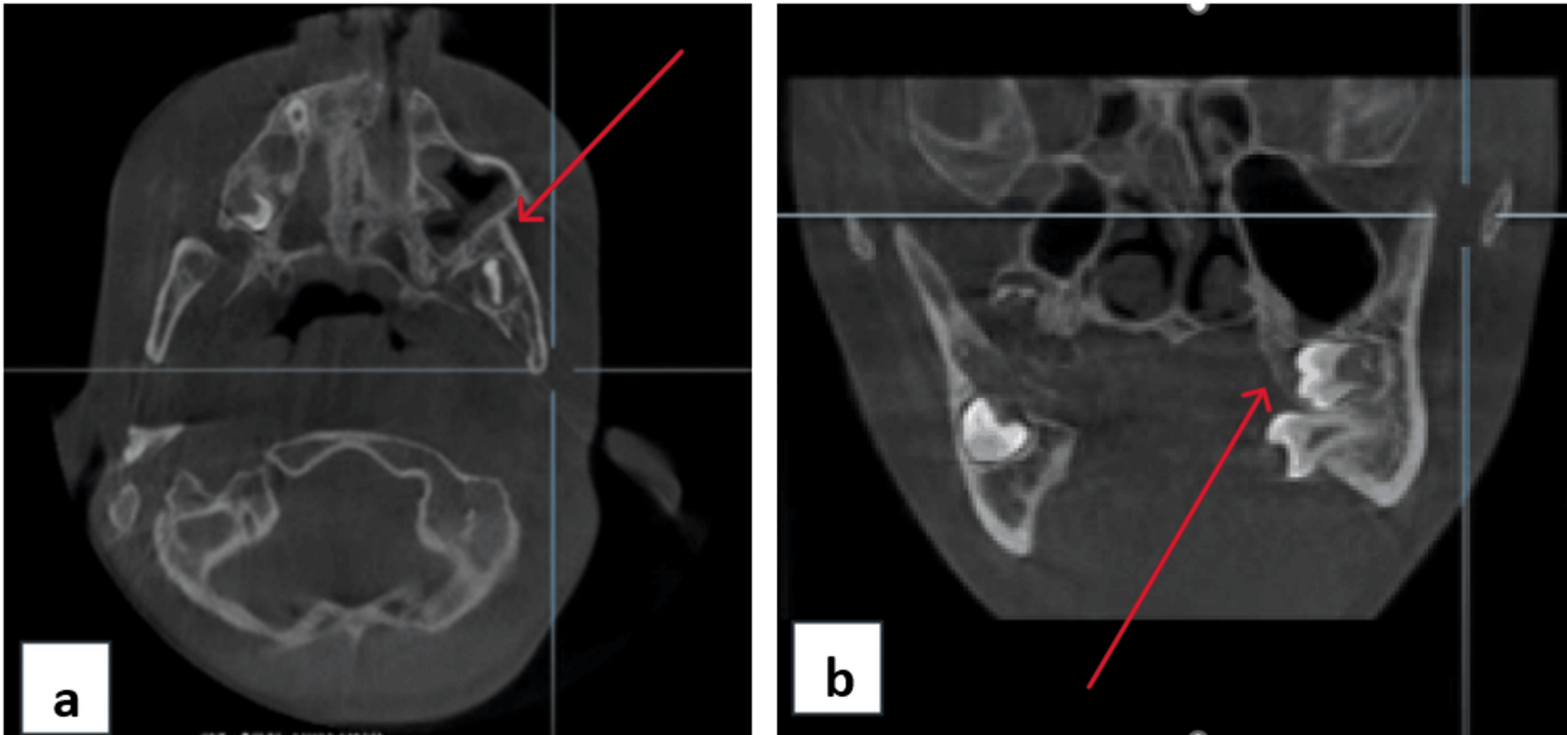
(a) Axial view shows bony fusion of the anterior border of the ramus of the mandible to that of the posterolateral wall of the maxillary sinus on the left side. (b) Coronal view shows widening of ramus on the left side and linguoversion of teeth due to reduced space and fusion of maxillary and mandibular arches distal to the maxillary premolar.

**Figure 4:**
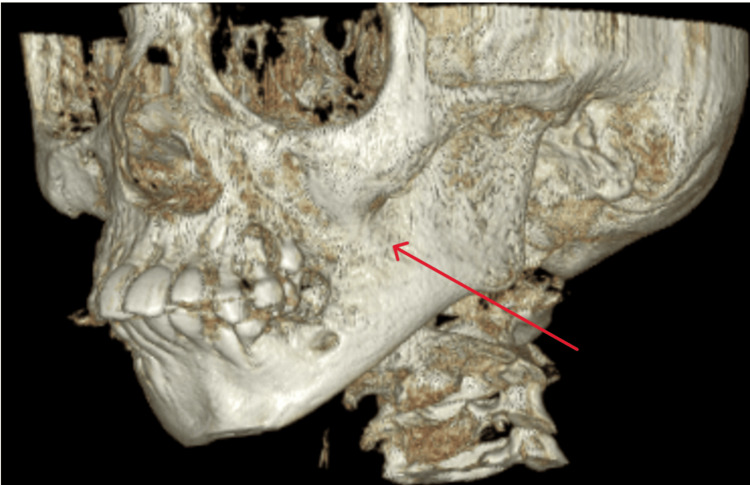
A 3D-rendered image revealed bony fusion on the left side, extending from the alveolar process of the mandible to the posterior region of the maxillary sinus and the zygomaticomaxillary complex.

A cone-beam computed tomography (CBCT) was advised, and it revealed bony fusion of the alveolar process of the maxilla and mandible, involving the zygomatic process of the maxilla extending till the posterolateral wall of the maxillary sinus. It showed no TMJ ankylosis, but narrowing of the joint space was seen bilaterally (Figure 3). A 3D-rendered image showed a clear extension of the fusion (Figure 4).

**Figure 5:**
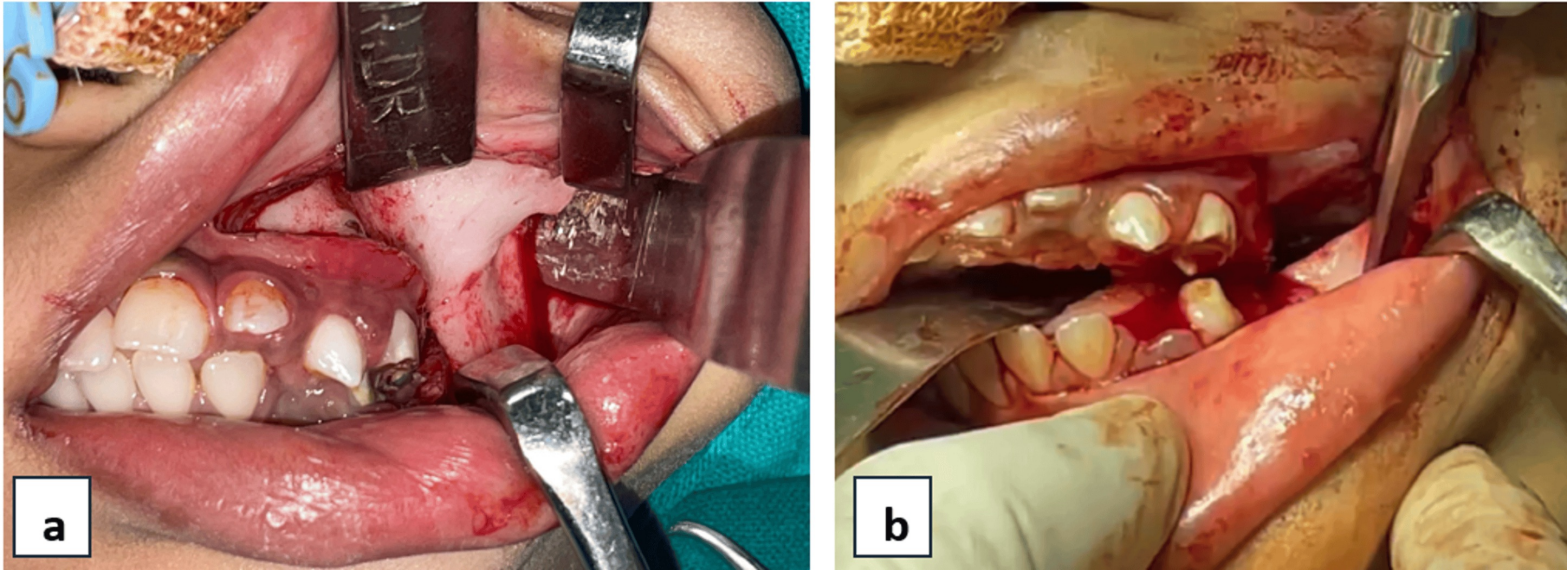
(a) Incision and exposure of bony fusion. (b) Osteotomy cuts were made using a microsaw to remove the bony mass.

Major surgical profile was advised, and pre-anesthetic clearance was obtained. Fiberoptic intubation was done due to zero mouth opening. Local anesthesia (2% lignocaine hydrochloride with 1:80,000 dilutions adrenaline) infiltrations were given at the sites where incision was planned. An incision extending from the lower vestibule, continuing onto the bony hard mass to the upper vestibule on the left side was given (Figure 5A). Osteotomy cuts were made using a reciprocating microsaw and chisels, and careful separation of the maxillary alveolus, tuberosity, maxillary sinus, and pterygoids was done from that of the bony mass. Similar osteotomy cuts were given in the mandible extending from the alveolus, body, and ascending ramus posteriorly. A 10 mm bony mass was removed after the osteotomy (Figure 5B).

**Figure 6:**
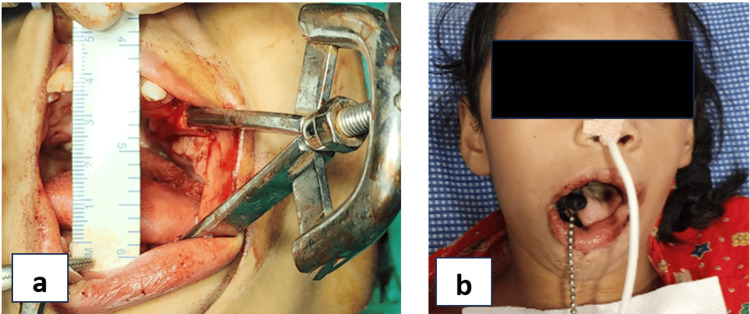
(a) Intraoperative mouth opening (36 mm). (b) Mouth opening was maintained using a bite block on the contralateral side.

**Figure 7:**
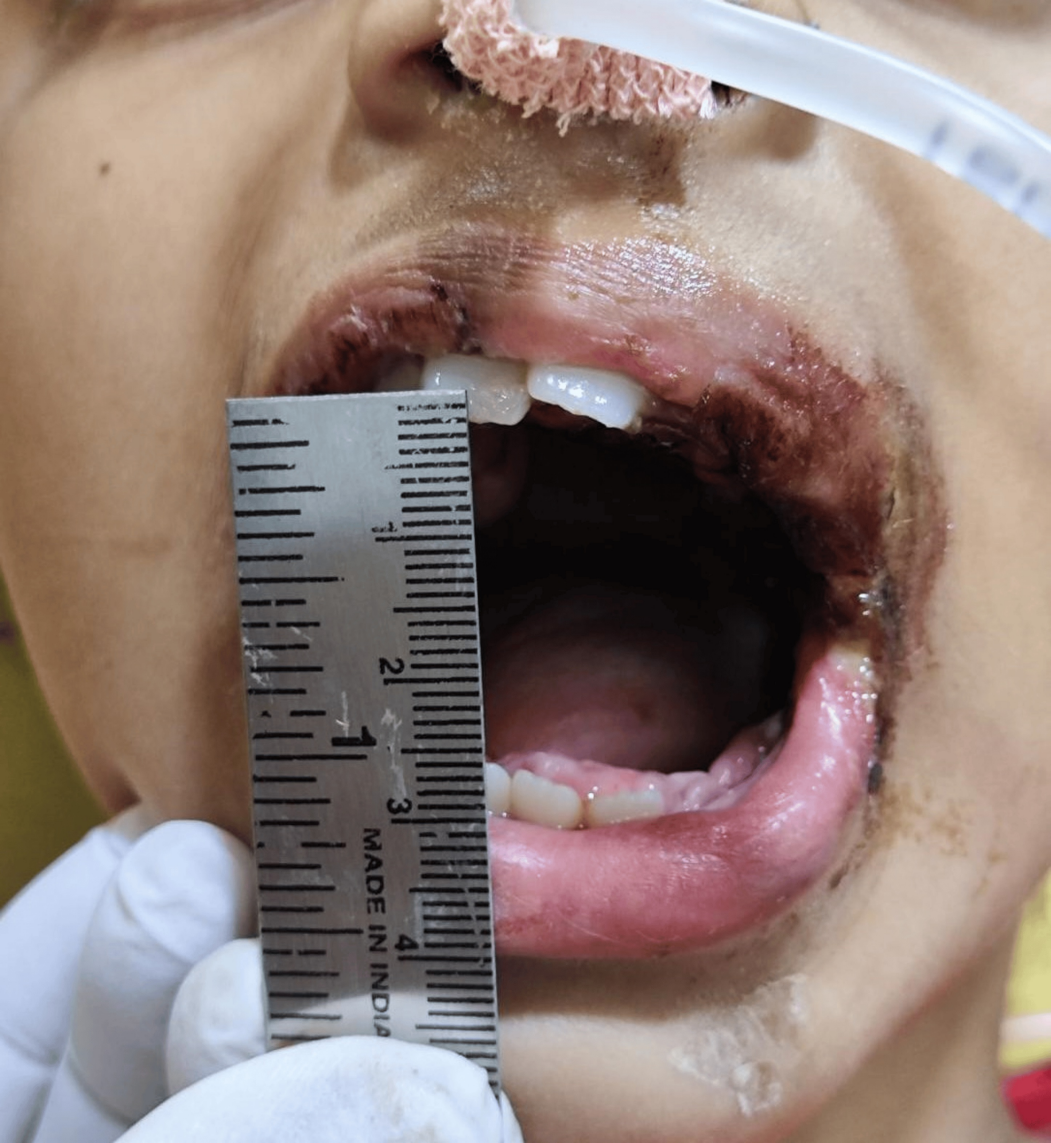
Mouth opening one week post operation.

To eliminate further resistance, masseter and medial pterygoid myotomy were done along with ipsilateral coronoidectomy, followed by closure of the raw surface using buccal fat pad, which was secured to the area using 3-0 Vicryl sutures. Postoperatively, after extubation, the mouth was kept open using a bite block on the contralateral side, and feeding was done through the nasogastric tube (Figure 6). The patient was discharged after a week, and the mouth opening measured at that time was 25 mm (Figure 7).

At one month follow-up, the patient had a complaint of mild swelling in the left side of the face and on intraoral examination, we noticed pus discharge from the operated site, which raised a suspicion of infected or necrotic bone, for which the intraoral site was opened and the necrotic bone was removed and metrogyl irrigation was done.

## Discussion

Fusion of the jaws and oral cavity represents a rare group of anomalies, with inconsistent terminology found throughout the literature. Various terms have been used to describe this condition, including zygomatico-mandibular fusion, congenital bony syngnathia, intra-alveolar synechiae, intraoral band, congenital fusion of the jaws or gums, and mandibulomaxillar synostosis, all referring to the severe limitation in mouth opening caused by either soft tissue or bony fusion of the jaws [2,3].

The exact cause of synechia or syngnathia remains unknown. However, several theories have been suggested by different authors to explain these rare malformations. Snijman and Prinsloo [4], for instance, propose that the condition results from an abnormal developmental process occurring during the later stages of intrauterine life. Various other experimental studies performed on the embryological basis include amniotic constriction bands in the region of the developing branchial arches, environmental insults, drugs such as meclozine, and large doses of vitamin A. Humphrey [5] has suggested that depressed fetal swallowing reflexes may delay palatal shelf elevation and this prolongs palatopharyngeal and palatoglossal epithelial contact leading to adhesion and cleft [6].

Several studies have identified real-time sonography as a reliable tool for assessing fetal movements, particularly facial expressions, during the second and third trimesters. These studies have documented fetal mouth opening associated with actions like swallowing, suckling, chewing, and even yawning in healthy fetuses. Continuous observation using real-time sonography (ranging from 15 minutes to two hours, depending on the study) has proven to be a consistent and reproducible method for visualizing mouth movements. Since fetuses with maxillomandibular fusion are unable to open their mouths, real-time sonography serves as a valuable diagnostic tool for ruling out this rare condition in suspected cases [7].

Clinical features seen in these cases are reduced mouth opening, causing difficulty in feeding and respiration. These findings are also sometimes associated with mandibular hypoplasia, glossopalatine ankylosis, oblique facial clefts, frontonasal malformations, tongue anomalies (small, bifid, or absent), hypophyseal duplication, clefts of the mandible, cleft palate, coloboma, and cleft lip [2]. This craniofacial anomaly is often associated with syndromes such as Van der Woude syndrome, popliteal pterygium syndrome, Dobrow syndrome, and craniofacial microsomia.

Numerous researchers have studied this condition, leading to the development of two classification systems for syngnathia: one proposed by Dawson and colleagues in 1997 [8], and another by Laster and associates in 2001 [9]. Dawson classified syngnathia into two types, of which type 1 is simple syngnathia. This type is defined by bony fusion between the mandible and maxilla or zygoma, with no other congenital anomalies present in the head and neck. Type 2 is complex syngnathia. This type involves bony fusion between the mandible and maxilla or zygoma, along with other congenital anomalies in the head and neck. Within this category, two subgroups are identified based on the associated conditions and the anatomical nature of the bony fusion: type 2a: syngnathia with aglossia; type 2b: syngnathia with agenesis or hypoplasia of the proximal mandible [8]. Laster’s modification of Dawson’s classification system includes the following categories: type 1a: simple anterior syngnathia involving bony fusion of the alveolar ridges only, without other congenital deformity in the head and neck; type 1b: complex anterior syngnathia identified by bony fusion of the alveolar ridges only and associated with other congenital deformity in the head and neck; type 2a: simple zygomatico-mandibular syngnathia characterized bony fusion of the mandible to the zygomatic complex causing only mandibular micrognathia; type 2b: complex zygomatico-mandibular syngnathia characterized by bony fusion of the mandible to the zygomatic complex, and associated with clefts or temporomandibular joint ankylosis [9].

While early intervention is often recommended to secure the airway, some reports have highlighted cases of bony refusion occurring after initial surgery, leading to the need for multiple procedures. There is no established management protocol for congenital syngnathia, and long-term functional outcomes following surgery are not extensively documented. Conversely, postponing the release of maxillomandibular fusion can present significant risks, including stunted growth, impaired development of the facial skeleton, aspiration pneumonia, asphyxia, abnormal eruption of teeth away from the alveolar ridge, and severe malnutrition due to delayed surgical intervention.

## Conclusions

This case report highlights the importance of early identification through comprehensive clinical and radiological evaluation. Surgical intervention remains the cornerstone of management, aiming to restore function and improve quality of life. Multidisciplinary collaboration among surgeons, anesthesiologists, and rehabilitation specialists is crucial for optimal outcomes. Continued research and case documentation are essential to enhance understanding and develop standardized protocols for this rare and complex condition.
